# Interim Estimates of 2025–26 Seasonal Influenza Vaccine Effectiveness — United States, September 2025–February 2026

**DOI:** 10.15585/mmwr.mm7509a2

**Published:** 2026-03-12

**Authors:** Patrick Maloney, Emily L. Reeves, Kristina Wielgosz, Ashley M. Price, Karthik Natarajan, Malini B. DeSilva, Kristin Dascomb, Nicola P. Klein, Sara Y. Tartof, Stephanie A. Irving, Shaun J. Grannis, Toan C. Ong, Zachary A. Weber, Jennifer E. Schuster, Danielle M. Zerr, Marian G. Michaels, Julie A. Boom, Natasha B. Halasa, Mary A. Staat, Geoffrey A. Weinberg, Stacey L. House, Elie A. Saade, Krissy Moehling Geffel, Manjusha Gaglani, Karen J. Wernli, Vel Murugan, Emily T. Martin, Natalie A. B. Bontrager, Marie K. Kirby, Amanda B. Payne, Fatimah S. Dawood, Ayzsa Tannis, Heidi L. Moline, Sifang Kathy Zhao, Katherine Adams, Jennifer DeCuir, Samantha M. Olson, Jessie R. Chung, Nathaniel Lewis, Brendan Flannery, Carrie Reed, Shikha Garg, Sascha Ellington, CDC Influenza Vaccine Effectiveness Collaborators, CDC Influenza Vaccine Effectiveness Collaborators, Omobosola Akinsete, Sarah W. Ball, Michelle A. Barron, Daniel Bride, Brian E. Dixon, John Hansen, David Mayer, Charlene McEvoy, Allison L. Naleway, Varsha Neelam, Caitlin Ray, Colin Rogerson, S. Bianca Salas, Tamara Sheffield, Lina S. Sy, Duck-Hye Yang, Ousseny Zerbo, Tara Curley, Kiran A. Faryar, Joanna Kramer, Jay Krishnan, Taylor Kristicevich, Aleda M. Leis, Amanda McKillop, Lora Nordstrom, Leah Odame-Bamfo, Ivana A. Vaughn, Emmanuel B. Walter, Elizabeth Wettick, Brianna M. Wickersham, Olivia L. Williams, Brian D. Williamson, Richard K. Zimmerman, Haris M. Ahmad, Christina Albertin, Vasanthi Avadhanula, James D. Chappell, Juliana DaSilva, Janet A. Englund, Megan C. Freeman, Lisa M. Keong, Eileen J. Klein, Mary E. Moffatt, Daniel C. Payne, Leila C. Sahni, Elizabeth P. Schlaudecker, Rangaraj Selvarangan, Laura S. Stewart, Peter G. Szilagyi, John V. Williams

**Affiliations:** ^1^Influenza Division, National Center for Immunization and Respiratory Diseases, CDC; ^2^Epidemic Intelligence Service, CDC; ^3^Department of Biomedical Informatics, Columbia University Irving Medical Center, New York, New York; ^4^Medical Informatics Services, New York-Presbyterian Hospital, New York, New York; ^5^HealthPartners Institute, Minneapolis, Minnesota; ^6^Division of Infectious Diseases and Clinical Epidemiology, Intermountain Health, Salt Lake City, Utah; ^7^Kaiser Permanente Vaccine Study Center, Kaiser Permanente Northern California Division of Research, Oakland, California; ^8^Department of Research & Evaluation, Kaiser Permanente Southern California, Pasadena, California; ^9^Kaiser Permanente Bernard J. Tyson School of Medicine, Pasadena, California; ^10^Kaiser Permanente Center for Health Research, Portland, Oregon; ^11^Center for Biomedical Informatics, Regenstrief Institute, Indianapolis, Indiana; ^12^School of Medicine, Indiana University, Indianapolis, Indiana; ^13^Department of Biomedical Informatics, University of Colorado Anschutz Medical Campus, Aurora, Colorado; ^14^Westat, Inc., Rockville, Maryland; ^15^Children’s Mercy Kansas City and University of Missouri—Kansas City School of Medicine, Kansas City, Missouri; ^16^Seattle Children’s Research Institute and University of Washington School of Medicine, Seattle, Washington; ^17^UPMC Children’s Hospital of Pittsburgh and University of Pittsburgh School of Medicine, Pittsburgh, Pennsylvania; ^18^Department of Pediatrics, Baylor College of Medicine and Texas Children’s Hospital, Houston, Texas; ^19^Department of Pediatrics, Vanderbilt University Medical Center, Nashville, Tennessee; ^20^Cincinnati Children’s Hospital Medical Center and Department of Pediatrics, University of Cincinnati College of Medicine, Cincinnati, Ohio; ^21^University of Rochester School of Medicine and Dentistry, Rochester, New York; ^22^Washington University School of Medicine, Department of Emergency Medicine, St. Louis, Missouri; ^23^University Hospitals Cleveland Medical Center, Case Western Reserve University School of Medicine, Cleveland, Ohio; ^24^University of Pittsburgh, School of Medicine, Department of Family Medicine, Pittsburgh, Pennsylvania; ^25^Baylor Scott & White Health, Department of Pediatrics, Temple, Texas; Baylor College of Medicine, Department of Pediatrics, Temple, Texas; ^26^Kaiser Permanente Washington Health Research Institute, Seattle, Washington; ^27^Arizona State University, Biodesign Center for Personalized Diagnostics, College of Health Solutions and Health Observatory, Tempe, Arizona; ^28^University of Michigan School of Public Health, Ann Arbor, Michigan; ^29^Duke University, Duke Human Vaccine Institute, Durham, North Carolina; ^30^Coronaviruses and Other Respiratory Viruses Division, National Center for Immunization and Respiratory Diseases, CDC.; HealthPartners Institute; Westat, Inc.; University of Colorado Anschutz Medical Campus; Intermountain Health; Regenstrief Institute; Kaiser Permanente Northern California; University of Colorado Anschutz Medical Campus; HealthPartners Institute; Kaiser Permanente Center for Health Research; National Center for Immunization and Respiratory Diseases, CDC; National Center for Immunization and Respiratory Diseases, CDC; Regenstrief Institute; Kaiser Permanente Southern California; Intermountain Health; Kaiser Permanente Southern California; Westat, Inc.; Kaiser Permanente Northern California.; Washington University School of Medicine in St. Louis; University Hospitals Cleveland Medical Center; Phoenix Children's Hospital; University Hospitals Cleveland Medical Center; Washington University School of Medicine in St. Louis; University of Michigan; Baylor Scott & White Health; Valleywise Health; Baylor Scott & White Health; Henry Ford Health; Duke Human Vaccine Institute; University of Pittsburgh Student Health Services; Kaiser Permanente Washington Health Research Institute; Duke Human Vaccine Institute; Kaiser Permanente Washington Health Research Institute; University of Pittsburgh School of Medicine.; National Center for Immunization and Respiratory Diseases,; CDC; University of Rochester School of Medicine and Dentistry; Baylor College of Medicine and Texas Children’s Hospital; Vanderbilt University Medical Center; National Center for Immunization and Respiratory Diseases, CDC; Seattle Children’s Research Institute; UPMC Children’s Hospital of Pittsburgh and University of Pittsburgh School of Medicine; National Center for Immunization and Respiratory Diseases, CDC; Seattle Children’s Research Institute; Children’s Mercy and University of Missouri-Kansas City School of Medicine; Cincinnati Children’s Hospital Medical Center and University of Cincinnati College of Medicine; Baylor College of Medicine and Texas Children’s Hospital; Cincinnati Children’s Hospital Medical Center and University of Cincinnati College of Medicine; Children’s Mercy and University of Missouri-Kansas City School of Medicine; Vanderbilt University Medical Center; University of Rochester School of Medicine and Dentistry and UCLA Department of Pediatrics; UPMC Children’s Hospital of Pittsburgh and University of Pittsburgh School of Medicine and University of Wisconsin School of Medicine and Public Health.

**Summary**

**What is already known about this topic?**

CDC routinely monitors influenza vaccine effectiveness (VE). Annual influenza vaccination is available for all eligible persons aged ≥6 months.

**What is added by this report?**

Interim 2025–26 seasonal influenza VE estimates were derived from three U.S. VE networks. Among children and adolescents, VE was 38%–41% against influenza-associated outpatient visits and 41% against influenza-associated hospitalization. Among adults aged ≥18 years, VE was 22%–34% against influenza-associated outpatient visits and 30% against influenza-associated hospitalization.

**What are the implications for public health practice?**

Receipt of a 2025–26 influenza vaccine reduced the risk for influenza-associated outpatient visits and hospitalizations. These findings support CDC’s influenza vaccination recommendations.

## Abstract

In the United States, annual influenza vaccination has been recommended for all persons aged ≥6 months, including during the 2025–26 season. Interim influenza vaccine effectiveness (VE) estimates were calculated for patients with acute respiratory illness–associated outpatient visits and hospitalizations from three U.S. respiratory virus VE networks during the 2025–26 influenza season, using a test-negative case-control design. Among children and adolescents aged <18 years, VE was 38%–41% against influenza outpatient visits and 41% against influenza-associated hospitalization. Among adults aged ≥18 years, VE was 22%–34% against influenza outpatient visits and 30% against influenza-associated hospitalization. Among children and adolescents, VE against influenza A ranged from 37% (against outpatient visits) to 42% (against hospitalization) across settings; among adults, VE against influenza A ranged from 30% (against hospitalization) to 34% (against outpatient visits) across settings. Among children and adolescents, VE against influenza A(H3N2)–associated outpatient visits was 35% and against influenza A(H3N2)–associated hospitalization was 38%. VE against influenza B outpatient visits ranged from 45%–71% among children and adolescents and was 63% among adults. Other estimates of VE were not statistically significant or were not reportable. Although interim influenza VE is lower during the 2025–26 influenza season than it was during recent influenza seasons, these findings demonstrate that influenza vaccination still provides protection against influenza. CDC recommends influenza vaccination; U.S. influenza vaccines remain available for persons aged ≥6 months.

## Introduction

CDC estimates that at least 26,000,000 illnesses, 340,000 hospitalizations, and 21,000 deaths resulting from influenza occurred in the United States during October 1, 2025–February 28, 2026. CDC’s Advisory Committee on Immunization Practices has recommended annual seasonal influenza vaccination for all persons aged ≥6 months, including during the 2025–26 influenza season (*1*). During the 2024–25 influenza season, influenza vaccination prevented an estimated 5 million medical visits, 180,000 hospitalizations, and 12,000 deaths caused by influenza viruses. During the current 2025–26 influenza season, 88% of subtyped influenza A–positive specimens were influenza A(H3N2); among those, 93% of genetically characterized samples were clade 2a.3a.1 subclade K (J.2.4.1), an antigenically drifted influenza virus that was first identified by CDC in June 2025 after selection of the 2025–26 vaccine viruses and that differs from the 2025–26 A(H3N2) influenza vaccine virus (*1*). Among sequenced influenza B viruses, 64% were clade C.3.1, which also differs antigenically from the 2025–26 influenza B vaccine virus. As of February 21, 2026, an estimated 48% of U.S. children and adolescents aged 6 months–17 years and 47% of U.S. adults aged ≥18 years had received an influenza vaccination during the 2025–26 season. CDC has monitored influenza vaccine effectiveness (VE) since 2004. This report provides interim estimates of 2025–26 influenza VE against outpatient visits and hospitalization for laboratory-confirmed influenza among children, adolescents, and adults from three U.S. surveillance networks.

## Methods

### Data Source and Collection

Data from three CDC-affiliated surveillance networks were used to assess influenza VE during September 2025–February 2026: 1) the New Vaccine Surveillance Network (NVSN), 2) the U.S. Flu VE Network (U.S. Flu VE), and 3) the Virtual SARS-CoV-2, Influenza, and Other respiratory viruses Network (VISION). Patient ages and care settings differ by network (Box). U.S. Flu VE enrolls only outpatients, NVSN* enrolls both outpatients and inpatients, and VISION retrospectively analyzes medical records of eligible outpatients and inpatients. All three networks include children and adolescents; U.S. Flu VE and VISION also include adults.

BOX **Characteristics of three influenza vaccine effectiveness networks — United States, 2025–26 influenza season**

**New Vaccine Surveillance Network (NVSN)**

- **Population:** Children and adolescents aged 6 months–17 years
- **Settings:** Outpatient (outpatient clinics, urgent care clinics, primary care offices, and emergency departments) and inpatient
- **Inclusion dates:** September 20, 2025–February 6, 2026
- **Type of surveillance:** Primarily active*
- **Medical centers (state):** Children’s Mercy (Missouri), University of Rochester Medical Center (New York), Cincinnati Children’s Hospital Medical Center (Ohio), UPMC Children’s Hospital of Pittsburgh (Pennsylvania), Vanderbilt University Medical Center (Tennessee), Texas Children’s Hospital (Texas), and Seattle Children’s Hospital (Washington)
- **Determination of influenza vaccination status:** Jurisdictional immunization registries, medical records, or parent/guardian/self-report
- **Acute respiratory illness (ARI) definition:** Symptoms of ARI (e.g., cough, fever, or other symptoms) ≤10 days of illness onset
- **Influenza A subtype available:** Yes

**U.S. Flu Vaccine Effectiveness Network**

- **Population:** Children and adolescents aged 8 months–17 years and adults aged ≥18 years
- **Settings:** Outpatient (outpatient clinics, urgent care clinics, and emergency departments)
- **Inclusion dates:** September 28, 2025–January 23, 2026
- **Type of surveillance:** Active
- **Medical centers (state):** Arizona State University Tempe, Phoenix Children’s Hospital, and Valleywise Health Medical Center (Arizona); University of Michigan and Henry Ford Health (Michigan); Washington University in St. Louis (Missouri); University Hospitals Cleveland Medical Center (Ohio); University of Pittsburgh and University of Pittsburgh Medical Center (Pennsylvania); Baylor Scott and White Health (Texas); and Kaiser Permanente Washington (Washington)
- **Determination of influenza vaccination status:** Jurisdictional immunization registries, medical records, or parent/guardian/self-report
- **ARI definition:** Illness ≤7 days in duration with new or worsening cough
- **Influenza A subtype available:** Yes

**Virtual SARS-CoV-2, Influenza, and Other respiratory viruses Network**

- **Population:** Children and adolescents aged 6 months–17 years and adults aged ≥18 years
- **Settings:** Outpatient (urgent care clinics and emergency departments) and inpatient
- **Inclusion dates:** October 1, 2025–January 23, 2026
- **Type of surveillance:** Passive
- **Medical centers (state):** Kaiser Permanente Northern California and Kaiser Permanente Southern California (California), UCHealth (Colorado), Regenstrief Institute (Indiana), HealthPartners (Minnesota and Wisconsin), and Kaiser Permanente Northwest (Oregon and Washington)
- **Determination of influenza vaccination status:** Jurisdictional immunization registries, electronic health records, and claims data
- **ARI definition:** Acute respiratory clinical diagnoses or respiratory signs or symptoms based on *International Classification of Diseases, Tenth Revision* codes
- **Influenza A subtype available:** No

* The majority of NVSN patients are actively enrolled. For this analysis, 1% of NVSN patients were passively enrolled.

Test-negative, case-control designs were used to estimate influenza VE among case-patients and control patients receiving outpatient or inpatient care for an acute respiratory illness (ARI) during the 2025–26 influenza season. ARI definitions varied by network. Case-patients were those with ARI who received a positive influenza virus molecular assay test result,^†^ and control patients were those with ARI who received a negative influenza virus test result.

### Data Analysis

To assess the association between influenza vaccination and influenza-associated outpatient visits or hospitalization, multivariable logistic regression was used. Odds ratios were calculated and adjusted for study site, patient age, date of illness, and other potential confounders.^§^ VE was estimated as (1 − adjusted odds ratio) × 100(%). Patients were considered vaccinated if they had received ≥1 dose of any 2025–26 influenza vaccine ≥14 days before the index date (ARI onset or clinical encounter).^¶^ Patients were excluded if they were vaccinated <14 days before the index date or had received a positive SARS-CoV-2 molecular test result at the time of testing for influenza** (*2*).

VE against an outpatient medical encounter and against hospitalization was calculated for any influenza, influenza A and B, and influenza A subtypes (A[H1N1]pdm09 and A[H3N2]) across networks and care settings, when possible. VE point estimates and 95% CIs are included in this report; CIs that exclude zero were considered statistically significant. VE estimates were not reported for strata with sparse data resulting in unstable model estimates, indicated by very wide CIs (range ≥100), even when case count thresholds were met (*3*).

Analyses were conducted using SAS software (version 9.4; SAS Institute) and R (version 4.5; R Foundation). NVSN and U.S. Flu VE activities were reviewed by CDC, deemed not research, and were conducted consistent with applicable federal law and CDC policy.^††^ VISION activities were reviewed by CDC, deemed not research or research not involving human subjects, and were conducted consistent with applicable federal law and CDC policy.^§§^

## Results

The analysis sample comprised 142,494 persons from all three surveillance networks, including 3,692 (3%) from NVSN, 3,380 (2%) from U.S. Flu VE, and 135,422 (95%) from VISION (Table 1) (Supplementary Table 1). Among the 3,692 children and adolescents aged 6 months–17 years included in NVSN, 2,296 (62%) were enrolled from outpatient settings, and 1,396 (38%) were hospitalized (Table 1). U.S. Flu VE included 3,380 outpatients aged ≥8 months. Among 135,422 VISION encounters for persons aged ≥6 months, 108,539 (80%) were outpatient and 26,883 (20%) were inpatient encounters.

**TABLE 1:** Number and percentage of patients who received medical care for an acute respiratory illness, by outpatient or inpatient setting, surveillance network, age group, and influenza test result — United States, 2025–26 influenza season

Network and patient age group (N = 142,494)	Outpatient setting*			Inpatient setting (hospitalization)		
	Total	Influenza test result, no. (row %)		Total	Influenza test result, no. (row %)	
		Positive (case-patients)	Negative (control patients)		Positive (case-patients)	Negative (control patients)
**NVSN**						
6 mos–17 yrs	**2,296**	729 (32)	1,567 (68)	**1,396**	248 (18)	1,148 (82)
**U.S. Flu VE**	**3,380**	731	2,649	**—**	—	—
8 mos–17 yrs	**1,069**	289 (27)	780 (73)	**—**	—	—
≥18 yrs	**2,311**	442 (19)	1,869 (81)	**—**	—	—
18–64 yrs	**1,784**	358 (20)	1,426 (80)	**—**	—	—
≥65 yrs	**527**	84 (16)	443 (84)	**—**	—	—
**VISION**	**108,539**	21,801	86,738	**26,883**	2,038	24,845
6 mos–17 yrs	**31,232**	8,253 (26)	22,979 (74)	**1,272**	93 (7)	1,179 (93)
≥18 yrs	**77,307**	13,548 (18)	63,759 (82)	**25,611**	1,945 (8)	23,666 (92)
18–64 yrs	**48,427**	9,973 (21)	38,454 (79)	**7,665**	639 (8)	7,026 (92)
≥65 yrs	**28,880**	3,575 (12)	25,305 (88)	**17,946**	1,306 (7)	16,640 (93)

**Abbreviations:** NVSN = New Vaccine Surveillance Network; U.S. Flu VE = U.S. Flu Vaccine Effectiveness Network; VISION = Virtual SARS-CoV-2, Influenza, and Other respiratory viruses Network.

* Outpatients received care in outpatient clinics, urgent care centers, primary care offices, and emergency departments (NVSN and U.S. Flu VE); and urgent care and emergency departments (VISION). Patients enrolled as outpatients in NVSN might have progressed to a more acute level of care; those data might not be reflected in this analysis.

### Influenza Vaccination Status

Among patients aged 6 months–17 years (NVSN and VISION) and 8 months–17 years (U.S. Flu VE) evaluated in outpatient settings, 17%–26% of case-patients and 22%–31% of control patients had received an influenza vaccination; among those who were hospitalized, 20%–33% of case-patients and 29%–43% of control patients had received an influenza vaccination (Table 2). Among adults evaluated in outpatient settings, 26%–32% of case-patients and 35%–40% of control patients had been vaccinated. Among those who were hospitalized, 37% of case-patients and 40% of control patients had received an influenza vaccination. Among adults aged ≥65 years evaluated in outpatient settings, 48%–61% of case-patients and 54%–68% of control patients had been vaccinated; 44% of case-patients and 46% of control patients who were hospitalized had received an influenza vaccination.

**TABLE 2:** Influenza vaccine effectiveness* among patients who received a 2025–26 influenza vaccine, by age group,^†^ percentage of patients vaccinated, influenza test result, and influenza type and subtype^§^ — United States, 2025–26 influenza season

Age group, influenza virus assessed, and network	Outpatient setting^¶^					Inpatient setting (hospitalization)				
	Influenza test result				VE (95% CI)**	Influenza test result				VE (95% CI)**
	Positive (case-patients)		Negative (control patients)			Positive (case-patients)		Negative (control patients)		
	Total	No. vaccinated (%)	Total	No. vaccinated (%)		Total	No. vaccinated (%)	Total	No. vaccinated (%)	
**All age groups**										
Any influenza^††^										
U.S. Flu VE	731	214 (29)	2,649	949 (36)	24 (8 to 38)	NA	NA	NA	NA	NA
VISION	21,801	4,943 (23)	86,738	27,338 (32)	36 (33 to 39)	2,038	735 (36)	24,845	9,779 (39)	31 (23 to 39)
**6 mos–17 yrs^†^**										
Any influenza^††^										
NVSN^§§^	729	149 (20)	1,567	485 (31)	41 (25 to 53)	248	81 (33)	1,148	490 (43)	41 (20 to 56)
U.S. Flu VE	289	74 (26)	780	193 (25)	14 (−20 to 39)	NA	NA	NA	NA	NA
VISION	8,253	1,432 (17)	22,979	4,958 (22)	38 (33 to 43)	93	19 (20)	1,179	339 (29)	48 (−12 to 78)
Influenza A										
NVSN^§§^	652	141 (22)	1,567	485 (31)	37 (20 to 50)	230	75 (33)	1,148	490 (43)	42 (21 to 57)
U.S. Flu VE	194	53 (27)	780	193 (25)	10 (−32 to 39)	NA	NA	NA	NA	NA
VISION	7,992	1,394 (17)	22,979	4,958 (22)	38 (33 to 43)	93	19 (20)	1,179	339 (29)	48 (−12 to 78)
Influenza A(H3N2)										
NVSN^§§^	598	132 (22)	1,567	485 (31)	35 (17 to 49)	187	63 (34)	1,148	490 (43)	38 (13 to 55)
U.S. Flu VE	147	43 (29)	780	193 (25)	2 (−49 to 37)	NA	NA	NA	NA	NA
Influenza B										
NVSN^§§^	80	8 (10)	1,567	485 (31)	71 (41 to 86)	19	7 (37)	1,148	490 (43)	—^¶¶^
U.S. Flu VE	95	21 (22)	780	193 (25)	20 (−41 to 56)	NA	NA	NA	NA	NA
VISION	272	39 (14)	22,979	4,958 (22)	45 (22 to 62)	—***	—***	1,179	339 (29)	—^¶¶^
**≥18 yrs**										
Any influenza^††^										
U.S. Flu VE	442	140 (32)	1,869	756 (40)	22 (1 to 39)	NA	NA	NA	NA	NA
VISION	13,548	3,511 (26)	63,759	22,380 (35)	34 (31 to 38)	1,945	716 (37)	23,666	9,440 (40)	30 (21 to 38)
Influenza A										
U.S. Flu VE	381	123 (32)	1,869	756 (40)	21 (−2 to 39)	NA	NA	NA	NA	NA
VISION	13,308	3,479 (26)	63,759	22,380 (35)	34 (30 to 37)	1,927	712 (37)	23,666	9,440 (40)	30 (21 to 38)
Influenza A(H3N2)										
U.S. Flu VE	295	103 (35)	1,869	756 (40)	11 (−18 to 33)	NA	NA	NA	NA	NA
Influenza B										
U.S. Flu VE	61	17 (28)	1,869	756 (40)	23 (−40 to 59)	NA	NA	NA	NA	NA
VISION	250	36 (14)	63,759	22,380 (35)	63 (48 to 75)	—***	—***	23,666	9,440 (40)	—^¶¶^
**18–64 yrs**										
Any influenza^††^										
U.S. Flu VE	358	89 (25)	1,426	455 (32)	24 (−1 to 42)	NA	NA	NA	NA	NA
VISION	9,973	1,778 (18)	38,454	8,832 (23)	36 (31 to 40)	639	135 (21)	7,026	1,710 (24)	29 (11 to 45)
Influenza A										
U.S. Flu VE	301	74 (25)	1,426	455 (32)	23 (−4 to 43)	NA	NA	NA	NA	NA
VISION	9,759	1,756 (18)	38,454	8,832 (23)	35 (30 to 39)	627	134 (21)	7,026	1,710 (24)	28 (8 to 43)
Influenza A(H3N2)										
U.S. Flu VE	232	64 (28)	1,426	455 (32)	12 (−22 to 37)	NA	NA	NA	NA	NA
Influenza B										
U.S. Flu VE	57	15 (26)	1,426	455 (32)	—^†††^	NA	NA	NA	NA	NA
VISION	220	24 (11)	38,454	8,832 (23)	66 (50 to 78)	—***	—***	7,026	1,710 (24)	—^¶¶^
**≥65 yrs**										
Any influenza^††^										
U.S. Flu VE	84	51 (61)	443	301 (68)	41 (1 to 64)	NA	NA	NA	NA	NA
VISION	3,575	1,733 (48)	25,305	13,548 (54)	30 (24 to 36)	1,306	581 (44)	16,640	7,730 (46)	31 (21 to 39)
Influenza A										
U.S. Flu VE	80	49 (61)	443	301 (68)	40 (−2 to 64)	NA	NA	NA	NA	NA
VISION	3,549	1,723 (49)	25,305	13,548 (54)	30 (24 to 36)	1,300	578 (44)	16,640	7,730 (46)	31 (20 to 40)
Influenza A(H3N2)										
U.S. Flu VE	63	39 (62)	443	301 (68)	37 (−14 to 65)	NA	NA	NA	NA	NA
Influenza B										
U.S. Flu VE	4	2 (50)	443	301 (68)	—^¶¶^	NA	NA	NA	NA	NA
VISION	30	12 (40)	25,305	13,548 (54)	—^¶¶^	—***	—***	16,640	7,730 (46)	—^¶¶^

**Abbreviations**: NA = not applicable; NVSN = New Vaccine Surveillance Network; OR = odds ratio; U.S. Flu VE = U.S. Flu Vaccine Effectiveness Network; VE = vaccine effectiveness; VISION = Virtual SARS-CoV-2, Influenza, and Other respiratory viruses Network.

* VE was estimated using the test-negative design comparing odds of receipt of 2025–26 influenza vaccination among persons with an acute respiratory illness who received a negative influenza or SARS-CoV-2 test result. Adjusted ORs were estimated using logistic regression; VE was calculated as (1 − adjusted OR) × 100(%). Firth logistic regression was used for estimates from NVSN.

^†^ Age group = 6 months–17 years (NVSN and VISION) and 8 months–17 years (U.S. Flu VE). All estimates were adjusted for geographic region, age, and date of illness. VISION also adjusted for sex and race and ethnicity.

^§^ Influenza A subtype was not available for VISION.

^¶^ Outpatients received care in outpatient clinics, urgent care centers, primary care offices, and emergency departments (NVSN and U.S. Flu VE) and in urgent care and emergency departments (VISION).

** CIs that exclude zero are considered statistically significant.

^††^ As of February 28, 2026, most influenza viruses detected were influenza A viruses (93%). Weekly US Influenza Surveillance Report: Key Updates for Week 8, ending February 28, 2026 | FluView | CDC

^§§^ Patients enrolled as outpatients in NVSN might have progressed to a more acute level of care; those data might not be reflected in this analysis.

^¶¶^ Estimates were not reported if there were fewer than 50 cases.

*** For VISION, cells with counts <5 were suppressed.

^†††^ Estimates were not reported if VE CI range was ≥100.

### VE Among Pediatric Patients

Among children and adolescents, VE against an outpatient visit for any influenza ranged from 38% (VISION) to 41% (NVSN); for influenza A, VE ranged from 37% (NVSN) to 38% (VISION); and for influenza B, VE ranged from 45% (VISION) to 71% (NVSN) (Table 2). VE against an outpatient visit for influenza A(H3N2) was 35% (NVSN). In U.S. Flu VE, estimates of VE against an outpatient visit for any influenza (14%), influenza A (10%), influenza A(H3N2) (2%), and influenza B (20%) were not statistically significant. VE against hospitalization for any influenza was 41%, for influenza A was 42%, and for influenza A(H3N2) was 38% (NVSN). In VISION, VE against hospitalization for any influenza and influenza A (48%) was not statistically significant. VE against hospitalization for influenza B was not reportable because of unstable model estimates resulting from sparsity of data. 

### VE Among Adult Patients

**Adults aged ≥18 years.** Among all adults aged ≥18 years, influenza VE against an outpatient visit for any influenza ranged from 22% (U.S. Flu VE) to 34% (VISION), for influenza A was 34% (VISION), and for influenza B was 63% (VISION). In U.S. Flu VE, estimates of influenza VE against an outpatient visit for influenza A (21%), influenza A(H3N2) (11%), and influenza B (23%) were not statistically significant. In VISION, influenza VE against hospitalization for any influenza and influenza A was 30%. VE against hospitalization for influenza B was not reportable.

**Adults aged 18–64 years.** Among adults aged 18–64 years in VISION, influenza VE against an outpatient visit for any influenza was 36%, for influenza A was 35%, and for influenza B was 66%. In U.S. Flu VE, estimates of VE against an outpatient visit for any influenza (24%), influenza A (23%), and influenza A(H3N2) (12%) were not statistically significant. In VISION, VE against hospitalization for any influenza was 29% and for influenza A was 28%. Influenza B VE against outpatient visits (U.S. Flu VE) and hospitalization (VISION) were not reportable.

**Adults aged ≥65 years.** Among adults aged ≥65 years, influenza VE against an outpatient visit for any influenza ranged from 30% (VISION) to 41% (U.S. Flu VE). VE against an outpatient visit for influenza A was 30% (VISION). In U.S. Flu VE, estimates of VE against an outpatient visit for influenza A (40%) and influenza A(H3N2) (37%) were not statistically significant. VE against hospitalization for any influenza and influenza A was 31% (VISION). VE estimates for influenza B were not reportable.

### Genetic Characterization of Influenza Viruses

As of February 18, 2026, a total of 572 influenza A(H3N2) viruses had been genetically characterized at CDC. Subclade K was detected in 474 (83%) samples (Supplementary Table 2) and was antigenically distinct from the 2025–26 A(H3N2) influenza vaccine virus (A/Croatia/10136RV/2023). Among 47 sequenced A(H1N1)pdm09 viruses and 108 sequenced B/Victoria viruses, clades D.3.1 (51%) and C.3.1 (81%) predominated, respectively. The B/Victoria clade C.3.1 differs from the influenza vaccine virus in the 2025–26 influenza season, whereas circulating A(H1N1) pdm09 viruses are similar to the selected vaccine virus (*1*).

## Discussion

Receipt of a 2025–26 seasonal influenza vaccine reduced the risk for influenza-associated outpatient visits (VE = 24%–36%) and influenza-associated hospitalization (VE = 31%) across all age groups. VE against outpatient visits was, on average, highest in children and adolescents (VE = 38%–41%). Protection against influenza-associated hospitalization was also highest in children and adolescents (VE = 41%), whereas VE against hospitalization in adults aged 18–64 years (VE = 29%) and ≥65 years (VE = 31%) was lower. Lower VE among older adults compared with that in younger persons has been observed in past influenza seasons, especially against A(H3N2) viruses (*4*).

Overall U.S. interim VE estimates against outpatient visits are comparable to estimates from China (24%), Canada (38% [influenza A]), and Europe (37%–40%) (*5*–*7*). U.S. interim estimates of VE against hospitalization are also comparable to estimates of VE in hospital settings in Europe (21%–42%) (*7*).

Sequencing data indicate that most influenza viruses circulating in the United States are A(H3N2) subclade K, an antigenically drifted virus predominant in the current season in most countries with available VE estimates. Lower influenza VE has been observed in some seasons when antigenically drifted viruses have circulated, but influenza vaccines have also been determined to provide protection against drifted viruses in previous recent influenza seasons (*4*). During the 2018–19 influenza season, a drifted A(H3N2) virus circulated, and an overall VE of 29% was observed (*8*).

U.S. interim VE estimates against influenza B outpatient visits are higher than estimates from Europe (−10%–35%) (*7*). VE against influenza B was high in both children and adults, consistent with the 2019–20 influenza season in the United States, during which influenza B viruses circulated and were antigenically drifted from the 2019–20 influenza B vaccine virus (*9*).

Influenza vaccination reduced the likelihood of both influenza-associated outpatient visits and hospitalizations, even with a circulating influenza A(H3N2) virus that is antigenically drifted from the vaccine virus. These findings support CDC’s recommendation for annual influenza vaccination (*1*). While influenza viruses continue to circulate, vaccination of eligible persons who have not yet received a 2025–26 influenza vaccine could reduce influenza-associated morbidity and mortality. CDC will continue to monitor influenza VE throughout the 2025–26 influenza season.

### Limitations

These findings are subject to at least four limitations. First, VE results are preliminary, and end-of-season estimates might change as influenza continues to spread during the 2025–26 season. Second, because factors such as underlying medical conditions and prior season influenza vaccination status were not modeled, the potential for unmeasured confounding exists. Third, self-reported vaccination data, receipt of vaccinations outside the medical system that were not documented, and the recommendation for administration of 2 influenza vaccine doses for children aged 6 months–8 years the first time they are vaccinated could have resulted in misclassification of vaccination status and biased VE estimates downward. Finally, small sample sizes precluded estimation of VE across all strata and VE against influenza A(H1N1)pdm09. Differences in VE estimates across networks might be the result of differences in ARI case definitions, active versus passive surveillance approaches that determine influenza testing practices and vaccination ascertainment (i.e., inclusion of self-report), outpatient care settings, and underlying site populations.

### Implications for Public Health Practice

As of February 21, 2026, fewer than one half of U.S. adults and children had received a 2025–26 influenza vaccine. Influenza vaccination can prevent medically attended illnesses and serious disease that might result in hospitalization or death. Even in seasons when overall VE is reduced, influenza vaccination has prevented thousands of hospitalizations and deaths, as observed in the 2022–23 influenza season when influenza VE was just 30%, yet influenza vaccines prevented an estimated 71,000 hospitalizations and 4,300 deaths. CDC recommends that eligible persons receive influenza vaccination (*1*); U.S. influenza vaccines remain available for persons aged ≥6 months. Influenza antiviral medications are an additional important public health tool, particularly during seasons with lower VE. Administration of antiviral medication is recommended as soon as possible for any patient with suspected or confirmed influenza who is hospitalized; has severe, complicated, or progressive illness; or is at higher risk for influenza complications. Receipt of a 2025–26 influenza vaccine reduced the risk for influenza-associated outpatient visits and hospitalizations.

## Footnotes

* Patients included as outpatients in NVSN might have progressed to a more acute level of care; those data might not be reflected in this analysis.

† To reduce potential case misclassification, all influenza case-patients received a positive reverse transcription–polymerase chain reaction test result from a clinical or surveillance respiratory laboratory specimen for NVSN and U.S. Flu VE. For VISION, influenza case-patients received a positive molecular assay result from a clinical respiratory laboratory specimen.

§ VISION also adjusted for sex and race and ethnicity.

¶ NVSN and U.S. Flu VE used date of ARI onset. VISION used the earliest of the following dates: outpatient visit, hospital admission, or influenza clinical testing.

** VISION also excluded patients who received a negative influenza test result but a clinical diagnosis of influenza, patients who received a clinical diagnosis of COVID-19, influenza case-patients who received a positive molecular test result for respiratory syncytial virus, and patients with missing information on sex.

†† 45 C.F.R. part 46.102(l)(2), 21 C.F.R. part 56; 42 U.S.C. Sect. 241(d); 5 U.S.C. Sect. 552a; 44 U.S.C. Sect. 3501 et seq.

§§ 45 C.F.R. part 46, 21 C.F.R. part 56; 42 U.S.C. Sect. 241(d); 5 U.S.C. Sect. 552a; 44 U.S.C. Sect. 3501 et seq.

## References

[1] Grohskopf LA, Blanton LH, Ferdinands JM, Reed C, Dugan VG, Daskalakis DC. Prevention and control of seasonal influenza with vaccines: recommendations of the Advisory Committee on Immunization Practices—United States, 2025–26 influenza season. MMWR Morb Mortal Wkly Rep 2025;74:500–7. 10.15585/mmwr.mm7432a2 40879559 PMC12393693

[2] Doll MK, Pettigrew SM, Ma J, Verma A. Effects of confounding bias in coronavirus disease 2019 (COVID-19) and influenza vaccine effectiveness test-negative designs due to correlated influenza and COVID-19 vaccination behaviors. Clin Infect Dis 2022;75:e564–71. 10.1093/cid/ciac234 35325923 PMC9129127

[3] Greenland S, Mansournia MA, Altman DG. Sparse data bias: a problem hiding in plain sight. BMJ 2016;352:i1981. 10.1136/bmj.i1981 27121591

[4] Belongia EA, Simpson MD, King JP, Variable influenza vaccine effectiveness by subtype: a systematic review and meta-analysis of test-negative design studies. Lancet Infect Dis 2016;16:942–51. 10.1016/S1473-3099(16)00129-8 27061888

[5] Shen Y, Zhang D, Feng Z, Modest protection from vaccination against influenza A(H3N2) subclade K, Beijing, China, 2025/26 season. Euro Surveill 2026;31:2600096. 10.2807/1560-7917.ES.2026.31.7.2600096 41716086 PMC12923999

[6] Separovic L, Sabaiduc S, Zhan Y, Interim 2025/26 influenza vaccine effectiveness estimates with immuno-epidemiological considerations for A(H3N2) subclade K protection, Canada, January 2026. Euro Surveill 2026;31:2600068. 10.2807/1560-7917.ES.2026.31.5.2600068 41645799 PMC12881843

[7] Lucaccioni H, Marques DF, Kirsebom F, ; European IVE group. Influenza vaccine effectiveness from nine studies during drifted A(H3N2) subclade K predominance, Europe, September 2025 to January 2026. Euro Surveill 2026;31:2600109. 10.2807/1560-7917.ES.2026.31.7.2600109 41716088 PMC12924001

[8] Flannery B, Kondor RJG, Chung JR, Spread of antigenically drifted influenza A(H3N2) viruses and vaccine effectiveness in the United States during the 2018–2019 season. J Infect Dis 2020;221:8–15. 10.1093/infdis/jiz543 31665373 PMC7325528

[9] Tenforde MW, Kondor RJG, Chung JR, Effect of antigenic drift on influenza vaccine effectiveness in the United States–2019–2020. Clin Infect Dis 2021;73:e4244–50. 10.1093/cid/ciaa1884 33367650 PMC8664438

